# Surgical Approach Influences the Risk of Intraoperative Periprosthetic Femoral Fracture in Primary Total Hip Arthroplasty: A Retrospective Cohort Study

**DOI:** 10.7759/cureus.106065

**Published:** 2026-03-29

**Authors:** Jorge A Izquierdo, Regina Diaz, Carlos Rafael Luna Lizarraga, Karen Sclar, Javier Camacho-Galindo

**Affiliations:** 1 Orthopaedics and Traumatology, Centro Medico American British Cowdray (ABC), Mexico, MEX; 2 Joint Surgery, Instituto Nacional de Rehabilitación Luis Guillermo Ibarra Ibarra, Mexico, MEX

**Keywords:** anterior hip approach, anterolateral hip approach, intraoperative periprosthetic fracture, lateral hip approach, open surgical approach, periprosthetic femoral fracture (pff), posterolateral hip approach, total hip arthroplasty (tha)

## Abstract

Background: Intraoperative periprosthetic femoral fracture is an uncommon but clinically relevant complication of total hip arthroplasty (THA). While several patient-related risk factors have been described, the role of surgical approach remains incompletely understood.

Methods: A retrospective cohort study was conducted, including 1,384 patients who underwent primary THA with an uncemented femoral component between January 2019 and March 2025 at a tertiary-care hospital. Surgical approaches were categorized as anterior, anterolateral, direct lateral, and posterolateral. The primary outcome was the occurrence of intraoperative femoral periprosthetic fracture. Fractures were classified using the Vancouver intraoperative classification, the Unified Classification System for Periprosthetic Fractures (UCPF), and the Mallory classification. Multivariable logistic regression analysis was performed to evaluate the association between surgical approach and fracture risk.

Results: A total of 50 intraoperative periprosthetic femoral fractures were identified, corresponding to an incidence of 3.6%. The anterolateral (odds ratio (OR) 2.46, 95% CI 1.07-5.66, p = 0.03) and direct lateral (OR 2.47, 95% CI 1.14-5.34, p = 0.02) approaches were associated with a higher likelihood of fracture compared with the posterolateral approach. The anterior approach was not significantly associated with fracture risk (OR 1.32, 95% CI 0.58-3.01, p = 0.50). Most fractures were stable metaphyseal patterns, predominantly classified as Vancouver A2 (39/50, 78%), UCPF B1 (35/50, 70%), and Mallory type I (21/40, 52.5%). The majority occurred during femoral canal preparation or final stem implantation and were primarily managed with cerclage fixation.

Conclusions: Surgical approach was associated with differences in the occurrence of intraoperative periprosthetic femoral fracture in primary THA. The anterolateral and direct lateral approaches demonstrated higher fracture rates compared with the posterolateral approach. However, given the retrospective observational design and the potential for residual confounding, these findings should be interpreted with caution and do not establish a causal relationship.

## Introduction

Total hip arthroplasty (THA) is one of the most successful procedures in orthopedic surgery and continues to increase worldwide. Projections estimate that the number of primary THA procedures will increase by approximately 71% by 2030, while revision procedures are expected to increase between 43% and 70% in the United States alone [1-3]. The excellent functional outcomes and improvements in quality of life associated with this procedure have led THA to be described as the “operation of the century” [4]. As the number of procedures continues to rise, understanding the complications associated with THA and the factors that contribute to their development has become increasingly important.

Intraoperative periprosthetic femoral fractures are an uncommon but clinically relevant complication of primary THA. These fractures may compromise the primary stability of the femoral component, increase operative time, and potentially affect implant survival by increasing the risk of early loosening or revision surgery [5,6]. In addition to their clinical implications, the management of these fractures frequently requires additional implants or surgical interventions, increasing healthcare costs and patient morbidity. Several patient-related factors have been associated with the development of intraoperative periprosthetic femoral fractures, including advanced age, female sex, and reduced bone quality [7,8]. In addition, anatomical characteristics of the proximal femur, such as cortical thickness, canal diameter, and overall femoral morphology, may influence implant fit and load distribution, potentially increasing susceptibility to fracture. However, surgical technique-related factors have also been increasingly recognized as important contributors to this complication.

The surgical approach used during THA plays a critical role in femoral exposure, soft-tissue tension, and the mechanical axis through which femoral canal preparation and implant insertion are performed. The most commonly used approaches include the direct anterior, anterolateral, direct lateral, and posterolateral approaches. Each technique has specific advantages and limitations related to surgical exposure, soft-tissue preservation, and postoperative recovery. Previous studies have suggested that certain surgical approaches may be associated with a higher risk of intraoperative femoral fracture. In particular, lateral-based approaches and, in some reports, the anterior approach have been associated with increased fracture risk when compared with the posterolateral approach [9-11]. Proposed mechanisms include limited femoral exposure, increased tension of surrounding soft tissues, and deviation of femoral instrumentation toward the medial cortex during canal preparation and stem insertion. However, these mechanisms remain theoretical and have not been directly evaluated in most clinical studies.

Despite these observations, the relationship between surgical approach and the risk of intraoperative periprosthetic femoral fracture remains incompletely understood, particularly in cohorts where multiple surgical approaches are routinely performed within the same institution. Therefore, the aim of the present study was to evaluate the association between surgical approach and the risk of intraoperative femoral periprosthetic fracture in patients undergoing primary total hip arthroplasty with an uncemented femoral component [12]. Secondary objectives included describing fracture patterns according to the Vancouver intraoperative classification [13], the Unified Classification System for Periprosthetic Fractures (UCPF) [14], and the Mallory classification [15], as well as the treatments implemented and postoperative rehabilitation strategies used in patients who developed this complication.

## Materials and methods

**Figure 1:**
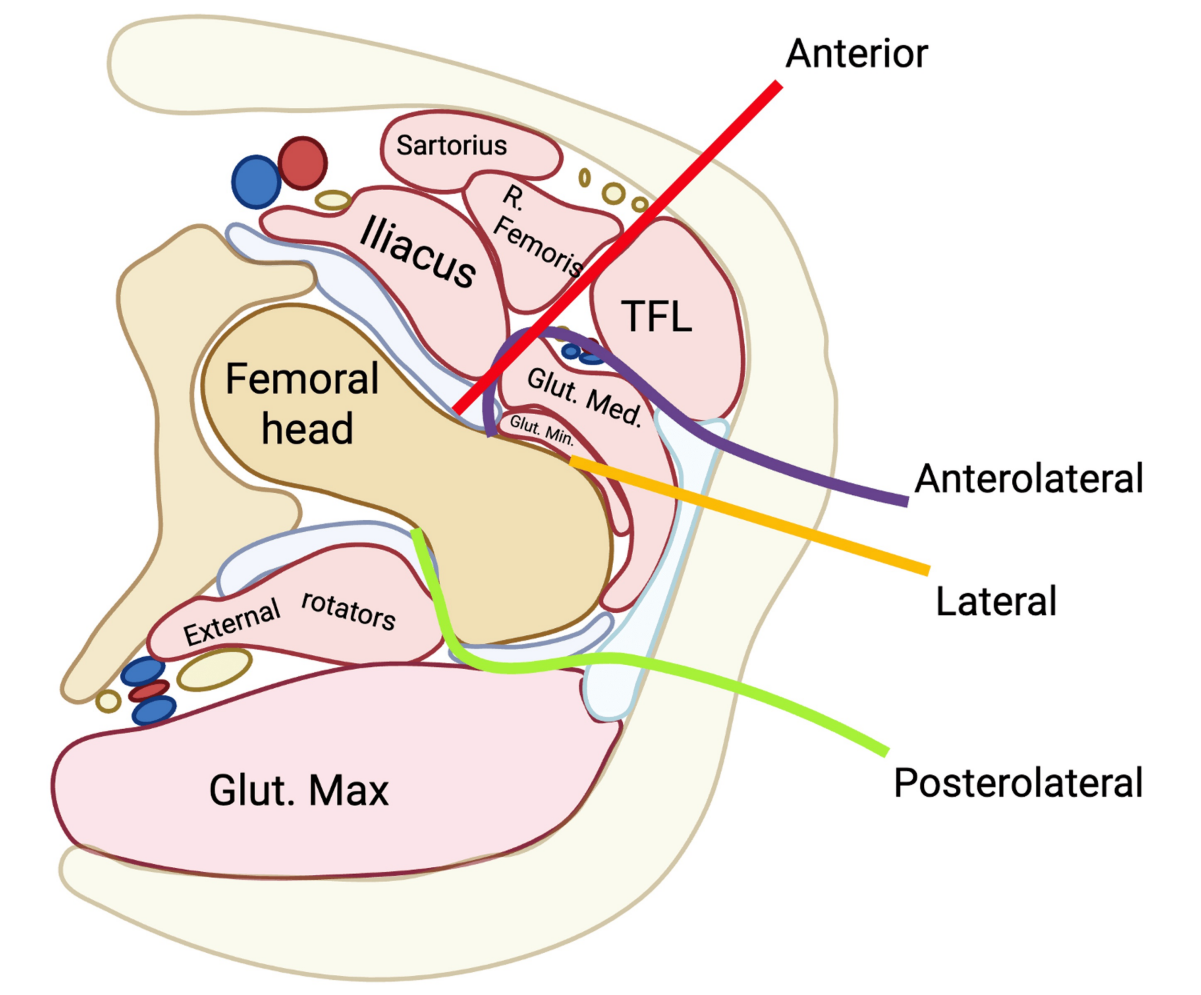
Schematic representation of the surgical approaches used in total hip arthroplasty. The anterior, anterolateral, direct lateral, and posterolateral approaches are illustrated relative to the proximal femur and surrounding soft tissues. Each trajectory reflects the typical direction of femoral canal access during implant preparation and insertion. Created with BioRender.com. No built-in AI-powered features were used in the creation of this figure. R. femoris = Rectus femoris; TFL = Tensor fasciae latae; Glut. med. = Gluteus medius; Glut. min. = Gluteus minimus; Glut. max. = Gluteus maximus.

This study was conducted as a retrospective cohort analysis of patients who underwent primary total hip arthroplasty (THA) with an uncemented femoral component between January 2019 and March 2025 at both hospital sites of Centro Médico American British Cowdray (ABC), a tertiary-care hospital system in Mexico City. The present investigation was derived from the same institutional cohort previously analyzed in a separate study evaluating the association between femoral stem geometry and intraoperative periprosthetic femoral fracture. However, the current analysis addresses a distinct research question focused on the influence of surgical approach on fracture risk. At the time of submission, the prior study derived from this cohort is under peer review. Figure 1 shows the common surgical approaches used in THA.

**Figure 2:**
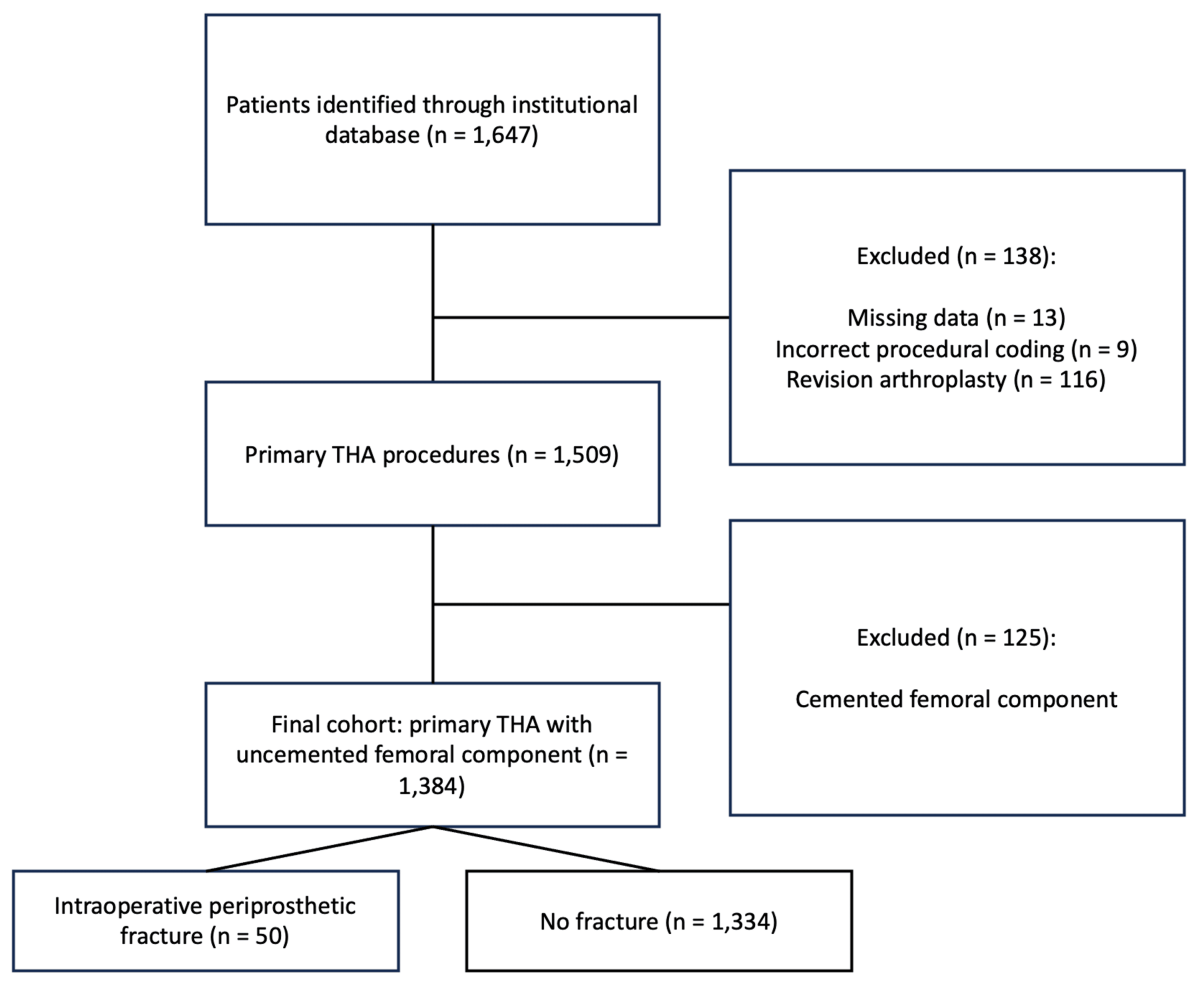
Flowchart of patient selection A total of 1,647 patients with procedural coding for hip arthroplasty were identified. After applying exclusion criteria, 1,384 patients undergoing primary total hip arthroplasty (THA) with an uncemented femoral component were included in the final analysis. The incidence of intraoperative periprosthetic femoral fracture was 3.6%. Image created by authors using Microsoft PowerPoint.

Patients were identified through the institutional surgical database using the International Classification of Diseases, Tenth Revision (ICD-10) coding system, specifically procedural codes corresponding to hip arthroplasty procedures (05R9, 05RA, 05RB, 05RE, 05RR, and 05R5) [12]. A total of 1,647 patients were initially identified. Patients were excluded if their medical records contained unrecoverable missing data, if the coded procedure did not correspond to hip arthroplasty, if the procedure corresponded to revision arthroplasty, or if the femoral component had been implanted using a cemented fixation technique. After applying these criteria, 1,384 patients undergoing primary THA with an uncemented femoral component were included in the final analysis (Figure 2).

Demographic and clinical variables collected from electronic medical records included age, sex, weight, height, body mass index, preoperative diagnosis, and comorbidities. Surgical variables included hospital site, surgical time, estimated blood loss, and length of hospital stay. The surgical approach used during the procedure was recorded and categorized as anterior, anterolateral, direct lateral, or posterolateral. Additional surgical variables relevant to the exposure were also collected. All procedures performed using the direct anterior approach were carried out using a traction table (HANA or Medacta) and with instrumentation specifically designed for the anterior approach, including offset broaching systems. Surgeon-related variables were also assessed, including the presence or absence of formal arthroplasty training. In this cohort, surgeons consistently used their preferred surgical approach for primary THA procedures.

The primary outcome of the study was the occurrence of intraoperative femoral periprosthetic fracture, defined as any femoral fracture identified during the surgical procedure or on immediate postoperative radiographs. In patients who developed this complication, fracture patterns were classified according to the intraoperative Vancouver classification [13], the Unified Classification System for Periprosthetic Fractures (UCPF) [14], and the Mallory classification [15]. Additional variables collected included the timing of fracture identification, treatment strategy used for fracture management, and postoperative rehabilitation protocols. Due to the retrospective nature of the study, the criteria guiding postoperative weight-bearing recommendations were not standardized and were determined at the discretion of the treating surgeon.

Statistical analysis was performed using SPSS Statistics software, version 27 (IBM Corp., Armonk, NY). Continuous variables were reported as mean and standard deviation or median and interquartile range, depending on data distribution, while categorical variables were expressed as frequencies and percentages. Comparisons between patients with and without fractures were performed using Student’s t-test or the Mann-Whitney U test for continuous variables and the Chi-square test for categorical variables.

Multivariable logistic regression analysis was performed to assess the association between surgical approach and intraoperative periprosthetic femoral fracture. Covariates were selected based on statistical significance in univariate analysis and clinical relevance and included age, sex, body mass index, preoperative diagnosis, surgical approach, and surgeon-related variables. The posterolateral approach was used as the reference category. Collinearity among variables was assessed using variance inflation factors. Additional analyses were performed to evaluate the robustness of the results. Statistical significance was defined as a p-value < 0.05. The study protocol was reviewed and approved by the institutional research and bioethics committee of Centro Médico ABC (approval no. CMABC-25-35). Given the retrospective design and the use of anonymized data, the study was considered minimal risk.

## Results

A total of 1,647 patients with procedural coding for hip arthroplasty between January 2019 and March 2025 were identified. After applying the inclusion and exclusion criteria, 1,384 patients who underwent primary total hip arthroplasty with an uncemented femoral component were included in the final analysis. Among these, 50 patients (3.6%) developed an intraoperative femoral periprosthetic fracture.

The median age of the overall cohort was 70 years (IQR 61-78), with 475 (34.3%) male and 909 (65.7%) female patients. Median body weight was 68 kg, median height was 165 cm, and median body mass index was 24.9 kg/m². In the fracture group, the median age was 69 years (IQR 60-78), with 34 (68.0%) female and 16 (32.0%) male patients. Median weight in this group was 66.5 kg, median height was 162 cm, and median body mass index was 25.0 kg/m². When comparing patients with and without fractures, the only variable that demonstrated a statistically significant difference was height, which was lower in the fracture group (162 cm vs 165 cm, p < 0.05) (Table 1).

**Table 1:** Baseline characteristics of the study population Continuous variables are presented as median and interquartile range (IQR), and categorical variables as number and percentage (%). Comparisons between groups were performed using the Student’s t test or Mann–Whitney U test for continuous variables, and the chi-square test for categorical variables. Statistical significance was defined as p < 0.05.

Variable	Total (N=1384)	No fracture (n=1334)	Fracture (n=50)	P value
Age (years), median (IQR)	70 (61–78)	70 (61–78)	69 (60–78)	0.51
Female sex, n (%)	909 (65.7)	875 (65.6)	34 (68.0)	0.73
BMI (kg/m²), median (IQR)	24.9 (22.8–27.6)	24.9 (22.8–27.6)	25.0 (22.8–27.6)	0.66
Diagnosis, n (%)				
Coxarthrosis	932 (67.3)	901 (67.5)	31 (62.0)	<0.05
Hip fracture	393 (28.4)	381 (28.6)	12 (24.0)	>0.05
Avascular necrosis	49 (3.5)	45 (3.4)	4 (8.0)	>0.05
Other	10 (0.7)	7 (0.5)	3 (6.0)	<0.05
Comorbidities, n (%)				
Hypertension	564 (40.8)	545 (40.9)	19 (38.0)	0.68
Diabetes mellitus	133 (9.6)	126 (9.4)	7 (14.0)	0.28
Osteoporosis	39 (2.8)	38 (2.8)	1 (2.0)	0.72

The most common preoperative diagnosis was coxarthrosis in 932 patients (67.3%), followed by hip fracture in 393 (28.4%), and avascular necrosis in 49 (3.5%). Logistic regression analysis demonstrated an increased risk of intraoperative periprosthetic femoral fracture in patients with sequelae of hip dysplasia compared with coxarthrosis (odds ratio (OR) 17.44, 95% CI 3.99-76.26). No other diagnoses were significantly associated with fracture occurrence. The most frequent comorbidities were systemic arterial hypertension in 564 patients (40.8%), thyroid disease in 245 (17.7%), and cardiac disease in 153 (11.1%). Osteoporosis was present in 39 patients (2.8%), of whom 1 (2.6%) developed an intraoperative fracture. No comorbidities demonstrated statistically significant differences between groups.

Regarding surgical approach, the anterior approach was used in 369 patients (26.7%), the anterolateral approach in 221 (16.0%), the direct lateral approach in 312 (22.5%), and the posterolateral approach in 482 (34.8%) (Table 2).

**Table 2:** Distribution of surgical approaches and fracture incidence Data are presented as numbers (%). Percentages for fracture and no fracture are calculated within each surgical approach group. P-value corresponds to the overall comparison between surgical approaches using the chi-square test. Statistical significance was defined as p < 0.05.

Surgical approach	Total (n, %)	No fracture (n, %)	Fracture (n, %)	P value
Anterior	369 (26.7)	359 (97.3)	10 (2.7)	
Anterolateral	221 (16.0)	209 (94.6)	12 (5.4)	
Direct lateral	312 (22.5)	295 (94.6)	17 (5.4)	
Posterolateral	482 (34.8)	471 (97.7)	11 (2.3)	
Overall comparison	—	—	—	0.03

The incidence of intraoperative periprosthetic fracture was 10 (2.7%) in the anterior group, 12 (5.4%) in the anterolateral group, 17 (5.4%) in the direct lateral group, and 11 (2.3%) in the posterolateral group (p = 0.03) (Table 3). Multivariable logistic regression analysis using the posterolateral approach as the reference demonstrated an increased likelihood of fracture for the anterolateral approach (OR 2.46, 95% CI 1.07-5.66, p = 0.03) and the direct lateral approach (OR 2.47, 95% CI 1.14-5.34, p = 0.02). The anterior approach was not significantly associated with fracture risk (OR 1.32, 95% CI 0.58-3.01, p = 0.50) (Table 3).

**Table 3:** Association between surgical approach and intraoperative periprosthetic femoral fracture Odds ratios (OR) were obtained using logistic regression analysis with the posterolateral approach as the reference category. Values are presented as OR with 95% confidence intervals (CI). Statistical significance was defined as p < 0.05.

Surgical approach	OR	95% CI	P value
Posterolateral	Ref	—	—
Anterior	1.32	0.58–3.01	0.50
Anterolateral	2.46	1.07–5.66	0.03
Direct lateral	2.47	1.14–5.34	0.02

Fractures were classified according to the intraoperative Vancouver classification, the Unified Classification System for Periprosthetic Fractures (UCPF), and the Mallory classification. The most frequent fracture pattern according to the Vancouver classification was type A2 in 39 patients (78.0%). According to the UCPF classification, the most common type was B1 in 35 patients (70.0%), while the Mallory classification most frequently identified type I fractures in 21 of 40 evaluated patients (52.5%) (Table 4).

**Table 4:** Fracture pattern distribution according to classification systems Data are presented as number (percentage). Percentages for each surgical approach are calculated within group. P values correspond to comparisons between surgical approaches for each classification system using the chi-square test. Statistical significance was defined as p < 0.05. UCPF= Unified Classification System for Periprosthetic Fractures

Classification	Total (N=50)	Anterior (n=10)	Anterolateral (n=12)	Direct lateral (n=17)	Posterolateral (n=11)	P value
Vancouver						0.03
A1	1 (2.0)	1 (10.0)	0 (0)	0 (0)	0 (0)	
A2	39 (78.0)	6 (60.0)	11 (91.7)	17 (100)	5 (45.5)	
A3	6 (12.0)	2 (20.0)	1 (8.3)	0 (0)	3 (27.3)	
B2	1 (2.0)	0 (0)	0 (0)	0 (0)	1 (9.1)	
B3	1 (2.0)	1 (10.0)	0 (0)	0 (0)	0 (0)	
C3	2 (4.0)	0 (0)	0 (0)	0 (0)	2 (18.2)	
UCPF						0.07
A1	5 (10.0)	2 (20.0)	1 (8.3)	2 (11.8)	0 (0)	
A2	1 (2.0)	1 (10.0)	0 (0)	0 (0)	0 (0)	
B1	35 (70.0)	5 (50.0)	10 (83.3)	15 (88.2)	5 (45.5)	
B2	5 (10.0)	1 (10.0)	1 (8.3)	0 (0)	3 (27.3)	
B3	2 (4.0)	1 (10.0)	0 (0)	0 (0)	1 (9.1)	
C	2 (4.0)	0 (0)	0 (0)	0 (0)	2 (18.2)	
Mallory (N=40)						0.51
Type I	21 (52.5)	3 (30.0)	5 (41.7)	9 (52.9)	4 (36.4)	
Type II	11 (27.5)	1 (10.0)	5 (41.7)	4 (23.5)	1 (9.1)	
Type III	8 (20.0)	2 (20.0)	1 (8.3)	2 (11.8)	3 (27.3)	

The most common intraoperative stages at which fractures were identified were during femoral canal preparation in 22 patients (44.0%) and during implantation of the definitive femoral component in 20 patients (40.0%). In three patients (6.0%), the fracture was identified on immediate postoperative radiographs (Table 5).

**Table 5:** Timing of intraoperative fracture identification Data are presented as number (percentage). Percentages are calculated within each surgical approach group. P value corresponds to the overall comparison between groups using the chi-square test. Statistical significance was defined as p < 0.05.

Timing of fracture identification	Total (N=50)	Anterior (n=10)	Anterolateral (n=12)	Direct lateral (n=17)	Posterolateral (n=11)	P value
Dislocation (traction table)	1 (2.0)	1 (10.0)	0 (0)	0 (0)	0 (0)	
Femoral osteotomy	2 (4.0)	1 (10.0)	0 (0)	1 (5.9)	0 (0)	
Femoral canal preparation	22 (44.0)	5 (50.0)	7 (58.3)	8 (47.1)	2 (18.2)	
Final stem implantation	20 (40.0)	0 (0)	0 (0)	1 (5.9)	0 (0)	
Reduction	12 (24.0)	2 (20.0)	5 (41.7)	7 (41.2)	7 (63.6)	
Postoperative control	3 (6.0)	2 (20.0)	0 (0)	0 (0)	1 (9.1)	
Unknown	1 (2.0)	0 (0)	0 (0)	0 (0)	1 (9.1)	
Overall comparison	—	—	—	—	—	0.20

Most fractures were managed with cerclage fixation. One cerclage was used in 24 patients (48.0%), and two cerclages were used in 15 patients (30.0%). Femoral stem revision was required in two patients (4.0%). In one patient (2.0%), the femoral component was revised to a cemented stem during the same procedure, and in one patient (2.0%), the fracture was identified postoperatively and treated during a second procedure with revision to a diaphyseal support tapered stem combined with five cerclages and a hook plate. Four patients (8.0%) did not receive surgical treatment for the fracture. Regarding postoperative management, 33 patients (2.4% of the total cohort) were instructed to delay weight-bearing in the immediate postoperative period. Among these, 10 patients (30.3%) had sustained an intraoperative fracture. In seven of these 10 patients (70.0%), the fracture had received appropriate surgical treatment, and six of these seven recommendations (85.7%) were made by surgeons with formal arthroplasty training.

## Discussion

In this study, 50 intraoperative periprosthetic femoral fractures were identified among 1,384 patients undergoing primary total hip arthroplasty with an uncemented femoral component, representing an overall incidence of 3.6%. This incidence remains within the wide range reported in the literature [6-19]. The primary objective of this study was to evaluate the association between surgical approach and the risk of intraoperative periprosthetic fracture. Our results demonstrated that both the anterolateral and direct lateral approaches were associated with a higher likelihood of fracture when compared with the posterolateral approach (OR 2.46 and OR 2.47, respectively). These findings are consistent with previous reports that have identified lateral-based approaches as potential risk factors for intraoperative femoral fracture [9-11].

One possible explanation for this association lies in the technical characteristics of these approaches. Both the anterolateral and direct lateral approaches involve partial detachment or manipulation of the abductor mechanism, which may alter femoral exposure and instrumentation alignment. This can lead to deviation of broaches and implants toward the medial cortex, increasing stress concentration and predisposing to fracture. In our cohort, 96% of fractures associated with these approaches were stable metaphyseal fractures, supporting this biomechanical hypothesis. However, it is important to emphasize that these explanations remain hypothetical, as these biomechanical variables were not directly measured in this study.

In contrast, the posterolateral approach, which served as the reference category in this study, demonstrated a lower observed fracture risk. This approach allows for more direct femoral access with less constraint from surrounding soft tissues, potentially facilitating more controlled canal preparation and implant insertion. Nevertheless, these findings should be interpreted cautiously, as differences in patient selection, surgeon preference, and procedural characteristics may also contribute to the observed associations.

Although the anterior approach has been associated with an increased risk of intraoperative fractures during the learning curve [9-11], this association was not observed in our study. This may be explained by variability in surgeon experience, instrumentation, and technique, as well as differences in patient selection. Importantly, the present study was not specifically designed to evaluate learning-curve effects, and detailed data regarding surgeon case volume and longitudinal experience were not systematically analyzed. Therefore, no definitive conclusions can be drawn regarding the relationship between the anterior approach and the learning curve.

An important aspect of this study is the evaluation of fracture patterns and their management. The majority of fractures identified were stable metaphyseal fractures, most commonly classified as Vancouver A2 [13], UCPF B1 [14], and Mallory type I [15]. These findings are consistent with previous literature describing medial cortical involvement as the most frequent pattern in intraoperative fractures [8,20]. The majority of these fractures occurred during femoral canal preparation or final implant insertion, further supporting the role of mechanical stress during press-fit fixation [5,8].

Regarding treatment, most fractures were managed with cerclage fixation, typically using one or two cables. This approach is consistent with current recommendations for stable metaphyseal fractures [13,15]. However, when analyzing treatment adequacy, a proportion of patients with more severe fracture patterns (Mallory type II or III) received suboptimal management, such as single cerclage fixation or inadequate positioning. Notably, most of these cases were associated with procedures performed by surgeons without formal arthroplasty training. However, given the design of the study, this observation should be interpreted cautiously and should not be considered as establishing a causal relationship between surgeon training and fracture management adequacy.

Interestingly, surgeon experience alone was not associated with a statistically significant difference in fracture incidence. However, the present study was not specifically designed to evaluate surgeon volume or learning curve effects, and therefore, this finding should be interpreted with caution. In our institution, surgeons without formal arthroplasty fellowship training often perform procedures with the assistance of experienced arthroplasty surgeons, which may partially mitigate differences in outcomes.

Postoperative rehabilitation strategies also differed between groups. Patients who developed intraoperative fractures were more likely to have restricted weight-bearing protocols. However, due to the retrospective nature of this study, postoperative rehabilitation protocols were not standardized and were determined at the discretion of the treating surgeon. Therefore, the criteria used to select immediate versus delayed weight-bearing could not be systematically evaluated, and no definitive conclusions can be drawn regarding optimal postoperative management. Although recent literature suggests that stable fractures may tolerate early weight-bearing, this was not specifically assessed in the present study [21,22].

The only baseline variable associated with fracture risk in this cohort was patient height, which was slightly lower in the fracture group. This finding may reflect anatomical differences in femoral structure, including canal size, cortical thickness, and implant fit; however, these parameters were not directly evaluated in this study due to incomplete radiographic data. A significant proportion of preoperative and postoperative imaging studies were performed outside our institution and were therefore not consistently available for analysis.

This study has several limitations. First, its retrospective observational design introduces the possibility of selection bias and information bias. Second, confounding by indication is likely present, as the choice of surgical approach may be influenced by patient characteristics, anatomical factors, and surgeon preference, all of which may also affect fracture risk. Third, residual confounding cannot be excluded due to the absence of important variables such as detailed femoral morphology, bone quality, implant characteristics, and comprehensive measures of surgeon experience.

Additional limitations include potential detection bias, as intraoperative fractures may have been identified with varying sensitivity across surgeons and approaches, particularly when postoperative imaging contributed to diagnosis. The lack of adjustment for clustering by surgeon or hospital site may also have influenced the observed associations. Furthermore, missing radiographic data limited the ability to evaluate anatomical risk factors in a consistent manner. Finally, temporal changes in surgical techniques, instrumentation, and surgeon experience over the study period may have introduced additional variability.

Despite these limitations, this study includes a large and heterogeneous cohort with a wide distribution of surgical approaches and implant types, reflecting real-world clinical practice. Additionally, the inclusion of fracture classification, treatment strategies, and rehabilitation protocols provides a comprehensive evaluation of this complication beyond simple incidence reporting. Overall, our findings suggest that the surgical approach was associated with differences in the occurrence of intraoperative periprosthetic femoral fracture in primary THA. However, given the observational design and the presence of potential confounding factors, these findings should be interpreted with caution and do not establish a causal relationship.

## Conclusions

The anterolateral and direct lateral approaches were associated with a higher likelihood of an intraoperative periprosthetic femoral fracture compared with the posterolateral approach, suggesting that differences in surgical exposure and instrumentation alignment may play a role in the development of this complication. Most fractures were stable metaphyseal patterns occurring during femoral preparation or stem insertion and were primarily managed with cerclage fixation.

Although surgeon training did not demonstrate a clear association with fracture incidence, it may have influenced fracture management patterns. However, given the design of this study, these observations should be interpreted with caution. Postoperative rehabilitation strategies varied, and delayed weight-bearing was more frequently recommended in patients with fractures, although criteria for these decisions were not standardized.

Overall, the surgical approach was associated with an increased risk of intraoperative periprosthetic femoral fracture in primary THA. However, the retrospective observational design and the presence of potential residual confounding preclude causal inference. These findings should therefore be interpreted with caution. Further prospective studies are required to better define the role of surgical approach and other contributing factors in the development of this complication.
